# Survey dataset on presenteeism, job demand and perceived job insecurity: The perspective of diplomatic officers

**DOI:** 10.1016/j.dib.2020.105505

**Published:** 2020-04-14

**Authors:** Daniella Mokhtar, Nurul-Azza Abdullah, Najwa Afiqa Roshaizad

**Affiliations:** Universiti Kebangsaan, Malaysia

**Keywords:** Presenteeism, Job demand, Job insecurity, Diplomatic officers

## Abstract

This data belongs to a sample of 347 diplomatic officers from two different locations which are the National Institute of Public Administration (INTAN) in Johor and Terengganu. This data consists of the demographic characteristics of the sample and three main variables. Each variable has sub-dimension which are (1) presenteeism: ability to complete a task and avoid disturbance, (2) job demand: workload, emotional and cognitive demand, (3) job insecurity: the importance and probability of an event to take place. The data were collected using a cross-sectional questionnaire via paper-and-pencil mode and was analyzed using SPSS version 22. Pearson correlation analyses revealed a significant relationship between avoiding disturbance (sub-dimension of presenteeism) and the probability of an event happening (sub-dimension of job insecurity) and no significant correlation between other variables. Analyses of the data can provide insights into determinants of presenteeism that may be useful for researchers in the field and policymakers that are interested in this area. It may serve as a reference to expand research and to develop interventions to facilitate productivity and well-being in the workplace.

Specifications table

**Table:** 

Subject	Applied Psychology, Psychology
Specific subject area	Industrial and Organizational Psychology, Occupational Psychology, Counterproductive Work Behavior (CWB)
Type of data	Table
How data were acquired	Data was acquired using a cross-sectional survey from 347 respondents. Survey consisted of demographic characteristics, presenteeism scale and perceive job insecurity.
Data format	Raw and descriptive
Parameters for data collection	Data was collected from diplomatic officers that was still undergoing training at the National Institute of Public Administration (INTAN).
Description of data collection	The research team went to the two locations at INTAN Terengganu & INTAN Johor to collect data via convenient sampling. Respondents were briefed on survey details and were data were collected via pencil and paper mode. Respondents were required to answer the survey on the spot and handed in their survey sheets at the end of the session.
Data source location	Institution: National Institute of Public Administration (INTAN) City/Town/Region: Kemaman, Terengganu (4.1954° N, 103.4407° E) & Kluang, Johor (2.0155° N, 103.2932° E) Country: Malaysia.
Data accessibility	The raw data files are provided in the Data in Brief Dataverse. All other data is with the article.

## Value of the data

• This data provides information on the work factors (i.e. work demand and job insecurity) with the presenteeism phenomena among diplomatic officers.
• The data on the work factors and level of presenteeism can be compared to other samples in a different job scope including health and education or a different sector such as the private sector.
• This dataset may help inform reliability and validity values on the adapted instruments.
• The data can be used by other researchers to further analyze other potential factors influencing presenteeism among employees.
• Considering the limited available data on presenteeism, this data set opens up an avenue for future research focused on factors influencing presenteeism as well as other research methods of researching presenteeism.

## 1 Data

The shared data includes response rates (Table 1), demographic data (Table 2) and descriptive values of variables measuring presenteeism, job demand and job insecurity (Table 3). A total of four hundred copies of questionnaire were administered to respondents from two National Institute of Public Administration located in Terengganu and Johor. According to Table 1, the total number of 390 questionnaires (97.50%) were returned and was filtered out for missing data. Forty-three questionnaires (10.75%) were removed due to missing data leaving a final of 347 usable questionnaires (86.75%) for analysis to take place. The demographic characteristics of the respondents are highlighted in Table 2 below. Meanwhile, Table 3 shows the descriptive statistics of the variables and its sub-dimensions. The raw data are shared as supplementary materials in two versions which are available in SPSS format and Excel format together with the data. The raw data includes demographic items (i.e. gender, age, race, religion, tenure, marital status, and education level), responses to each item, computed response including its dimension (total score) and scores based on it sub-dimension (eg. presenteeism as its total score, avoiding disturbance and ability to complete a task as the sub-dimensions of presenteeism). Levels of each dimension are also included, categorizing the dimensions into three levels; low, medium and high. Besides that, the survey questions are also included as supplementary material where it is available in two languages, Malay and English.

**Table 1:** Rate of response of the administered questionnaire.

Questionnaire		Number of respondents	Rate of response (%)
Administered	400		
Not Returned		10	2.50
Returned		390	97.50
Usable		347	86.75
Not usable		43	10.75
Total	400	400	100.00

*Source:* Researcher's Field Survey, 2014.

**Table 2:** Demographic characteristics of respondents.

Demographical Characteristics	Items	Frequency	Percentage (%)
**Gender**	Male	96	27.7
	Female	251	72.3
**Age**	25–30	249	71.8
	31–35	82	23.6
	36 and above	16	4.6
**Race**	Malay	314	90.5
	Chinese	16	4.6
	Indian	9	2.6
	Others	8	2.3
**Religion**	Muslim	317	91.4
	Christian	8	2.3
	Buddhist	13	3.7
	Hindu	8	2.3
	Others	1	.3
**Marital Status**	Single	97	28.0
	Married	244	70.3
	Divorced	6	1.7
**Education Background**	Degree	279	80.4
	Masters	67	19.3
	PhD	1	.3
**Years of Service**	Less than 1 year	8	2.3
	1–5 years	302	87.0
	6–10 years	26	7.5
	More than 10 years	11	3.2

*Source:* Researcher's Field Survey, 2014.

**Table 3:** Descriptive statistics of the variables contained in the dataset.

Variable	N	Minimum	Maximum	Mean		Std. Deviation
				Statistic	Std. Error	
Presenteeism	347	11	30	21.90	.19	3.50
Complete Task	347	3	15	10.98	.12	2.14
Avoid Disturbance	347	4	15	10.92	.12	2.25
Job Demand	347	30	76	48.57	.42	7.82
Workload	347	8	20	14.89	.12	2.30
Emotional Demand	347	8	28	18.59	.22	4.00
Cognitive Demand	347	7	28	15.09	.32	5.97
Job Insecurity	347	31	90	60.24	.56	10.40
Importance	347	9	45	33.76	.37	6.91
Probability	347	11	45	26.49	.36	6.70

*Source:* Researcher's Field Survey, 2014.

### 1.1 Relationship between all variables

Findings revealed that the first sub-dimension of presenteeism ‘ability to complete a task’ did not have any relationship with sub-dimensions of job demand; workload (*r* = 0.02, *p* > .05), emotional (*r* = 0.03, *p* > .05) and cognitive demand (*r* = 0.02, *p* > .05) as well as job insecurity; importance (*r* = −3, *p* > .05) and probability (*r* = −0.01, *p* > .05). Meanwhile, the second subdimension of presenteeism ‘avoid disturbance’ also show no significant relationship with sub-dimensions of job demand; workload (*r* = 0.05, *p* > .05), emotional (*r* = 0.04, *p* > .05) and cognitive demand (*r* = 0.03, *p* > .05). However, there was a significant relationship with one of the sub-dimensions of job insecurity which is the probability of an event taking place (*r* = −0.12, *p* < .05) but not with the importance of the event taking place (*r* = 0.01, *p* > .05). The event within the job insecurity context refers to potential events that could happen to one's current job. This includes losing one's job, transferred to a different job, pressured to accept an early retirement (Refer to supplementary material: job insecurity questionnaire).

## 2 Experimental design, materials, and methods

The data represents a quantitative study to assess the relationship between presenteeism, job demand and perceived job insecurity among employees. A cross-sectional survey method was used for data collection. Samples were among diplomatic officers from two National Institute of Public Administration located in Terengganu and Johor. Permission was obtained from the authorities from the Institutes where the research would take place. The questionnaire consisted of adapted items from previous research [1], [2], [3], [4], [5] and went through a back translation process to the Malay Language. The survey was divided into four sections which consisted of demographical questions (Section 1), presenteeism (Section 2), job demand (Section 3) and job insecurity (Section 4). Section 1 collects information on the demographical background of the respondents. Among the information that were gathered are gender, age, race, religion, level of education, tenure and marital status.

Section 2 consists of six items adapted from the short version of Stanford presenteeism Scale (SPS-6) which was built by Koopman and his colleagues in 2002. Presenteeism refers to a condition where individuals are present at work but not performing well where at the same time, they are supposed to be at home resting due to their illness or too many hours spent working. The variable is divided into two dimensions, namely ‘ability to complete a task’ and ‘avoid distractions’. The items are all positive for an example, ‘Even though I have health problems, I was able to complete the tasks in the workplace’. The scoring methods of this scale uses a 5-point Likert Scale with a continuum of 1-5 where 1: Strongly Agree, 2: Disagree. 3: Not Sure, 4: Agree and 5: Strongly Agree. The range of this scale is from a minimum score of 6 to a maximum score of 30. The higher the score obtained by the respondent, show a higher in presenteeism.

Section 3 consisted of 19 items; 5 items were adapted from Job Content Questionnaire (Karasek et al., 1985) while the other 14 items were adapted from the Experience and Evaluation of Work questionnaire (van Veldhoven and Mejimen, 1994). The scale is divided into three dimensions of job demands in terms of workload, emotional and cognitive aspects. The scoring methods of this scale uses a 4-point Likert Scale with a continuum of 1-4 where 1: Too Frequent, 2: Frequent, 3: Sometimes, and 4: Never. The range of this scale is from a minimum score of 19 to a maximum score of 76. The higher the score obtained by the respondent, shows a higher demand in their job. Lastly, Section 4 consists of 18 items that was adapted from the Job Insecurity Scale (Ashford, 1989). The items were divided into two categories which were perceived importance and perceived tendency of an unwanted event taking place. The scoring methods of the perceived importance items uses a 5-point Likert Scale with a continuum of 1(highly unimportant) to 5 (highly important) meanwhile for perceived tendency uses a 5-point Likert Scale with a continuum of 1 (highly impossible) to 5 (highly possible).

Respondents were informed on the background and purpose of the research. It was highlighted that their identity will remain anonymous and there are free to withdraw at any point emphasizing autonomy. They were then briefed on the instructions and a method example was demonstrated to increase their understanding. Apart from providing a verbal description, the questionnaires distributed also consist of written instructions that respondents can use as a reference. Once finished, they were then asked to answer the questionnaires on their own without giving any time limit. The respondents did not take more than 45 min and they were allowed to ask questions along the way. In general, they did not face any difficulties in answering the questions as it was asked in layman terms and simple sentences. Once completed, the questionnaires were collected by hand for further steps in the analysis. The data were keyed in manually into the Statistical Package for Social Sciences (SPSS) and were analyzed using descriptive and inferential statistics (Tables 1-4).

**Table 4:** Pearson's correlation values between the variables in the dataset.

Dimension	Sub-dimension	1a	1b	2a	2b	2c	3a	3b
1. Presenteeism	1a. Complete Task	–						
	1b Avoid Disturbance	.27**	–					
2. Job Demand	2a. Workload	.02	.05	–				
	2b. Emotional	.03	.04.	−0.28**	–			
	2c. Cognitive	.02	.03	−0.25**	.33**	–		
3. Job Insecurity	3a. Importance	−0.03	.01	.15*	−0.02	−0.31**	–	
	3b. Probability	−0.01	−0.12*	−0.07	.04	.05	.15	–

*Source:* Researcher's Field Survey, 2014.

## Appendix Supplementary materials

Image, application 1

Image, application 2

Image, application 3

Image, application 4

Image, application 5
