# Supplementary material for: Survey dataset on presenteeism, job demand and perceived job insecurity: The perspective of diplomatic officers
Source: Data Brief. 2020 Apr 14;30:105505. doi: 10.1016/j.dib.2020.105505 (PMC7186489; doi:10.1016/j.dib.2020.105505)
Supplement: Supplementary file 2 [file mmc2.docx]

**SURVEY ON PRESENTEEISM, JOB DEMAND & JOB INSECURITY**

**SECTION 1: Demographic Questions**

1. Gender : Male ( )

Female ( )

2. Age : …………… (years)

3. Marital Status : Single ( )

Married ( )

Divorced ( )

4. Race : Malay ( )

Chinese ( )

Indian ( )

Other Race ( )

Please state :………………..

5. Religion : Islam ( )

Christian ( )

Buddhist ( )

Hindu ( )

Other ( )

Please state :………………..

6. Tenure : Less than 01 year ( )

01 - 05 years ( )

06 - 10 years ( )

11 - 15 years ( )

16 - 20 years ( )

21 - 25 years ( )

26 years and above ( )

7. Highest Educationan Qualification : SPM

STPM/ Diploma/Certificate( )

Undergraduate ( )

Masters ( )

PhD ( )

Others ( )

Please state:………………..

**SECTION 2: Presenteeism**

This section contains 6 questions. Please tick (√) according to choices of answers given below.

**1** = Strongly disagree, **2** = Disagree, **3** = Neutral, **4** = Agree, **5** = Strongly Agree

| **No** | **Item** | **1** | **2** | **3** | **4** | **5** |
| --- | --- | --- | --- | --- | --- | --- |
| 1. | I find it difficult to manage work-related stress because of my health problems |  |  |  |  |  |
| 2. | I am able to complete my tasks despite my health problem |  |  |  |  |  |
| 3. | My health problems prevent me from enjoying my work |  |  |  |  |  |
| 4. | I give up trying to complete specific tasks because of my health problems |  |  |  |  |  |
| 5. | I am able to focus in achieving my goals at work even with my health problems |  |  |  |  |  |
| 6. | I’m feel energized in completing my work even with my health problems |  |  |  |  |  |

**SECTION 3: Job Demand**

This section contains 19 questions. Please tick (√ ) according to choices of answers given below.

**1** = Strongly disagree, **2** = Disagree, **3** = Agree, **4** = Strongly Agree

| **No** | **Item** | **1** | **2** | **3** | **4** |
| --- | --- | --- | --- | --- | --- |
| 1. | My job requires me to work fast |  |  |  |  |
| 2. | My job requires me to work hard |  |  |  |  |
| 3. | I was asked to not overworked myself |  |  |  |  |
| 4. | I have enough time to finish my tasks |  |  |  |  |
| 5. | I’m free from the conflicting demands from others. |  |  |  |  |

1 = Always, 2 = Most of the time, 3 = Sometimes, 4 = Never

| **No** | **Item** | | **1** | **2** | **3** | **4** |
| --- | --- | --- | --- | --- | --- | --- |
| 6. | Does your job require a lot of emotional demands? | |  |  |  |  |
| 7. | Does your job require a lot of concentration? | |  |  |  |  |
| 8. | Does your job involves dealing with things that effect you on a personal level? | |  |  |  |  |
| 9. | Does your job require you to be precise and accurate? | |  |  |  |  |
| 10. | | Do others call on you personally in your work? |  |  |  |  |
| 11. | | Do you need to concentrate on more than one thing at the same time? |  |  |  |  |
| 12. | | Do you feel personally threatened or attacked in your line of work? |  |  |  |  |
| 13. | | Does your work require you to continuously come out with new ideas? |  |  |  |  |
| 14. | | Do you deal with difficult clients or patients in your line of work? |  |  |  |  |
| 15. | | Does your work require you to concentrate at long intervals? |  |  |  |  |
| 16. | | Does your job require you to be able to convince or persuade other people? |  |  |  |  |
| 17. | | Does your line of work require you to memorize a lot of things? |  |  |  |  |
| 18. | | Does your work expose you to potential emotional pain? |  |  |  |  |
| 19. | | Does your work require you to be extra cautious? |  |  |  |  |

**SECTION 4: Job Insecurity**

This section contains 18 questions. Please tick (√ ) according to choices of answers given below.

Assume for a moment that each of the following events could happen to you in your current job.

Please indicate on one of the five alternatives how IMPORTANT to you personally is the possibility of each of the following event.

**1** = Very unimportant, **2** = Unimportant, **3** = Neutral, **4** = Important, **5** = Very Important

| **No** | **Item** | **1** | **2** | **3** | **4** | **5** |
| --- | --- | --- | --- | --- | --- | --- |
| 1. | You might lose your job and demoted to a lower position in the organization |  |  |  |  |  |
| 2. | You might lose your job and laterally transferred to another position in the organization |  |  |  |  |  |
| 3. | Your organization provides flexible amount of working hours daily for your particular job |  |  |  |  |  |
| 4. | You might be transferred to a different job with a higher position at your current geographical location |  |  |  |  |  |
| 5. | You might be transferred to a different job with a higher position at a different geographical location |  |  |  |  |  |
| 6. | Your services can be terminated permanently |  |  |  |  |  |
| 7. | Your department’s future is uncertain |  |  |  |  |  |
| 8. | You might be fired from your job. |  |  |  |  |  |
| 9. | You might be pressured to accept an early retirement |  |  |  |  |  |

Again, thinking about the future, HOW LIKELY is it that each of these events might actually occur to you in your current job.

Please indicate your answer on one of the five alternatives below each question.

**1** = Very unlikely, **2** = Unlikely, **3** = Neutral, **4** = Likely,

**5 =** Most Likely

| **No** | **Item** | **1** | **2** | **3** | **4** | **5** |
| --- | --- | --- | --- | --- | --- | --- |
| 10. | Lose your job and transferred to a lower level job in the organization |  |  |  |  |  |
| 11. | Lose your job and transferred to the same level job in the organization |  |  |  |  |  |
| 12. | Finding out that your total working hours offered by the organization can change from day to day |  |  |  |  |  |
| 13. | Being transferred to a higher-level position at your current location |  |  |  |  |  |
| 14. | Being transferred to a higher-level position at a different geographical location |  |  |  |  |  |
| 15. | Lose your job and be terminated permanently |  |  |  |  |  |
| 16. | Finding out your department/division’s future is uncertain |  |  |  |  |  |
| 17. | Losing your job for being fired. |  |  |  |  |  |
| 18. | Losing your job for being pressured to retire early |  |  |  |  |  |
