# Supplementary material for: Survey dataset on presenteeism, job demand and perceived job insecurity: The perspective of diplomatic officers
Source: Data Brief. 2020 Apr 14;30:105505. doi: 10.1016/j.dib.2020.105505 (PMC7186489; doi:10.1016/j.dib.2020.105505)
Supplement: Supplementary file 3 [file mmc3.docx]

KAJIAN SURVEI PRESENTEEISM, TUNTUTAN KERJA SERTA JAMINAN KERJA

**BAHAGIAN 1: Maklumat Demografi**

1. Jantina : Lelaki ( )

Perempuan ( )

2. Umur : …………… (tahun)

3. Status Perkahwinan : Bujang ( )

Berkahwin ( )

Janda/ Duda ( )

4. Bangsa : Melayu ( )

Cina ( )

India ( )

Lain-lain bangsa ( )

Nyatakan:………………..

5. Agama : Islam ( )

Kristian ( )

Buddha ( )

Hindu ( )

Lain-lain agama ( )

Nyatakan:………………..

6. Status Pekerjaan : Penjawat awam ( )

Penjawat swasta ( )

M48 ( )

M52 ( )

M54 ( )

7. Tempoh Perkhidmatan : Kurang 01 tahun ( )

01 - 05 tahun ( )

06 - 10 tahun ( )

11 - 15 tahun ( )

16 - 20 tahun ( )

21 - 25 tahun ( )

26 tahun dan ke atas ( )

8. Tahap Pendidikan Tertinggi : SPM

STPM/ Diploma/ Sijil

Ijazah sarjana muda ( )

Ijazah Sarjana ( )

PhD ( )

Lain-lain ( )

Nyatakan:………………..

9. Negeri : …………………………

**BAHAGIAN 2: PRESENTEEISM**

Bahagian ini mengandungi 6 soalan. Sila tandakan (√ ) berdasarkan pilihan jawapan di bawah.

**1** = Sangat tidak setuju, **2** = Tidak setuju, **3** = Neutral, **4** = Setuju, **5** = Sangat setuju

| **No** | **Soalan** | **1** | **2** | **3** | **4** | **5** |
| --- | --- | --- | --- | --- | --- | --- |
| 1. | Saya mengalami kesukaran dalam menangani tekanan dalam kerja disebabkan masalah kesihatan saya. |  |  |  |  |  |
| 2. | Walaupun saya mempunyai masalah kesihatan, saya mampu menyiapkan tugasan di tempat kerja. |  |  |  |  |  |
| 3. | Masalah kesihatan saya menganggu keseronokan saya dalam perkerjaan. |  |  |  |  |  |
| 4. | Saya berasa putus asa dalam menyiapkan tugasan tertentu disebabkan masalah kesihatan saya. |  |  |  |  |  |
| 5. | Di tempat kerja, saya mampu fokus dalam mencapai matlamat walaupun mempunyai masalah kesihatan. |  |  |  |  |  |
| 6. | Walaupun mempunyai masalah kesihatan, saya berasa bertenaga untuk menyiapkan semua kerja. |  |  |  |  |  |

**BAHAGIAN 3: TUNTUTAN KERJA**

Bahagian ini mengandungi 19 soalan. Sila tandakan (√ ) berdasarkan pilihan jawapan di bawah.

**1** = Sangat tidak setuju, **2** = Tidak setuju, **3** = Setuju, **4** = Sangat setuju

| **No** | **Soalan** | **1** | **2** | **3** | **4** |
| --- | --- | --- | --- | --- | --- |
| 1. | Pekerjaan saya memerlukan saya bekerja dengan pantas. |  |  |  |  |
| 2. | Pekerjaan saya memerlukan saya berkerja keras. |  |  |  |  |
| 3. | Saya tidak diminta untuk melakukan kerja yang berlebihan. |  |  |  |  |
| 4. | Saya mempunyai masa yang cukup untuk meyiapkan tugasan. |  |  |  |  |
| 5. | Saya bebas dari sebarang permintaan dari orang lain yang mendatangkan konflik. |  |  |  |  |

1 = Sangat selalu, 2 = Selalu, 3 = Kadang kala, 4 = Tidak pernah

| **No** | **Soalan** | | **1** | | **2** | **3** | **4** |
| --- | --- | --- | --- | --- | --- | --- | --- |
| 6. | Adakah kerja anda mengenakan banyak tuntutan emosi? | |  | |  |  |  |
| 7. | Adakah kerja anda menuntut banyak tumpuan? | |  | |  |  |  |
| 8. | Adakah anda berhadapan dengan perkara-perkara yang memberi kesan kepada anda secara peribadi dalam kerja anda? | |  | |  |  |  |
| 9. | Adakah kerja anda sentiasa memerlukan ketepatan? | |  | |  |  |  |
| 10. | | Adakah orang lain sering memanggil anda secara peribadi dalam kerja anda? | |  |  |  |  |
| 11. | | Adakah anda perlu menumpukan perhatian kepada banyak perkara pada masa yang sama? | |  |  |  |  |
| 12. | | Adakah anda rasa diserang atau diancam secara peribadi dalam kerja anda? | |  |  |  |  |
| 13. | | Adakah kerja anda memerlukan pemikiran berterusan? | |  |  |  |  |
| 14. | | Adakah anda berurusan dengan pelanggan yang sukar dalam kerja anda? | |  |  |  |  |
| 15. | | Adakah anda perlu memberi perhatian yang berterusan untuk kerja anda? | |  |  |  |  |
| 16. | | Dalam kerja-kerja anda, adakah anda perlu mampu meyakinkan atau memujuk orang? | |  |  |  |  |
| 17. | | Adakah anda perlu ingat banyak perkara dalam kerja anda? | |  |  |  |  |
| 18. | | Adakah kerja anda meletakkan anda dalam keadaan emosi yang menyakitkan? | |  |  |  |  |
| 19. | | Adakah kerja anda memerlukan anda untuk sentiasa berhati-hati? | |  |  |  |  |

**BAHAGIAN 4: JAMINAN KERJA**

Bahagian ini mengandungi 18 soalan. Sila tandakan (√ ) berdasarkan pilihan jawapan di bawah.

Anggap/ Bayangkan sekiranya keadaan tersebut akan terjadi pada pekerjaan anda sekarang. Pada pendapat anda, apakah kepentingan yang anda rasakan terhadap kemungkinan tersebut?

**1** = Sangat tidak penting, **2** = Tidak penting, **3** = Neutral, **4** = Penting, **5** = Sangat penting

| **No** | **Soalan** | **1** | **2** | **3** | **4** | **5** |
| --- | --- | --- | --- | --- | --- | --- |
| 1. | Anda mungkin kehilangan kerja anda dan diturunkan pangkat yang lebih rendah dalam organisasi. |  |  |  |  |  |
| 2. | Anda mungkin kehilangan kerja anda dan dipindahkan ke pekerjaan lain yang sama tahap dalam organisasi. |  |  |  |  |  |
| 3. | Syarikat menawarkan kerja yang fleksibel kepada anda. |  |  |  |  |  |
| 4. | Anda mungkin dinaikkan pangkat dan dipindahkan kepada pekerjaan lain di termpat kerja anda. |  |  |  |  |  |
| 5. | Anda mungkin dinaikkan pangkat dan dipindahkan ke pekerjaan yang berbeza di tempat lain. |  |  |  |  |  |
| 6. | Anda boleh diberhentikan secara kekal. |  |  |  |  |  |
| 7. | Masa depan bahagian atau jabatan anda ini mungkin tidak menentu. |  |  |  |  |  |
| 8. | Anda mungkin dipecat.*.* |  |  |  |  |  |
| 9. | Anda mungkin didesak untuk menerima persaraan awal. |  |  |  |  |  |

Sekali lagi, bayangkan masa depan anda. Apakah KEMUNGKINAN yang ada untuk situasi di bawah untuk berlaku dalam pekerjaan anda sekarang?

Nyatakan jawapan anda berdasarkan pilihan di bawah.

**1** = Sangat tidak mungkin, **2** = Tidak mungkin, **3** = Neutral, **4** = Berkemungkinan,

**5 =** Sangat berkemungkinan

| **No** | **Soalan** | **1** | **2** | **3** | **4** | **5** |
| --- | --- | --- | --- | --- | --- | --- |
| 10. | Kehilangan pekerjaan anda dan diturunkan pangkat di organisasi. |  |  |  |  |  |
| 11. | Kehilangan pekerjaan anda dan dipindahkan ke pekerjaan lain pada tahap yang sama dalam organisasi. |  |  |  |  |  |
| 12. | Mendapati bahawa bilangan jam yang boleh ditawarkan oleh syarikat anda untuk bekerja boleh berubah-ubah dari hari ke hari. |  |  |  |  |  |
| 13. | Dinaikkan pangkat dalam organisasi yang sama |  |  |  |  |  |
| 14. | Dinaikkan pangkt dan dipindahkan ke cawangan organisasi yang berbeza. |  |  |  |  |  |
| 15. | Kehilangan pekerjaan anda dan diberhentikan secara kekal. |  |  |  |  |  |
| 16. | Mendapati masa depan jabatan atau bahagian anda tidak menentu. |  |  |  |  |  |
| 17. | Kehilangan pekerjaan anda dengan dipecat. |  |  |  |  |  |
| 18. | Kehilangan pekerjaan anda dengan cara didesak untuk menerima persaraan awal. |  |  |  |  |  |

************Terima kasih kerana sudi meluangkan masa menjawab soal selidik ini************
